# A Snapshot of the Experience of Dietitians during the COVID-19 Crisis in Five Arab Countries: Findings from a Regional Cross-Sectional Study

**DOI:** 10.3390/nu14224904

**Published:** 2022-11-19

**Authors:** Khlood Bookari, Jamila Arrish, Sara Zaher, Majid Alkhalaf, Mudi Alharbi, Najla Alkhattaf, Ziad Harb, Eiman Al Hinai, Nahla Alanqodi, Sana Almajed, Maryam Aldwarji, Nawal Alqaoud, Jalila Elati, Maha Hoteit

**Affiliations:** 1 Department of Clinical Nutrition, Faculty of Applied Medical Sciences, Taibah University, Madinah 42353, Saudi Arabia; 2 National Nutrition Committee (NNC), Saudi Food and Drug Authority (Saudi FDA), Riyadh 13513, Saudi Arabia; 3 Healthcare Administration, Faculty of Applied Health Sciences, Saudi Electronic University, Riyadh 13316, Saudi Arabia; 4 Department of Nutrition, Faculty of Pharmacy, Saint Joseph University, Beirut P.O. Box 17-5208, Lebanon; 5 Dietetics and Nutrition Department, Al Nahdha Hospital, Ministry of Health, Muscat P.O. Box 937, Oman; 6 Dietetics and Nutrition Department, Sultan Qaboos University, Muscat P.O. Box 50, Oman; 7 Food and Nutrition Administration, Ministry of Health, Kuwait City 12009, Kuwait; 8 National Institute of Nutrition and Food Technology, Tunis 1007, Tunisia; 9 Faculty of Public Health, Lebanese University, Beirut P.O. Box 6573/14, Lebanon; 10 PHENOL Research Group Public Health Nutrition Program-Lebanon, Faculty of Public Health, Lebanese University, Beirut P.O. Box 6573/14, Lebanon; 11 Lebanese University Nutrition Surveillance Center (LUNSC), Lebanese Food Drugs and Chemical Administrations, Lebanese University, Beirut P.O. Box 6573/14, Lebanon

**Keywords:** COVID-19, dietitians, practices, duties, hospitals, clinics, Arab countries

## Abstract

COVID-19’s intimidating spread has challenged the resilience of the global health systems, causing shifts in the practices of healthcare workers, including dietitians. The current study aimed to assess the change in dietitians’ practices and duties in hospitals/clinics after the commencement of COVID-19 pandemic. This cross-sectional study was conducted in five Arab countries between November 2020 and January 2021. A convenient sample of 903 dietitians filled an online self-administered questionnaire to meet the study aims. Nearly 40.0% of the dietitians experienced a change in their workload and caseload during the pandemic. Besides, 18.7% of the dietitians had been assigned additional tasks in their facilities. Nearly half the dietitians (46.9%) had started giving remote nutrition consultations, associated with a 21% drop in the number of dietitians offering in-person consultations (*p* = 0.001). Approximately 58.9% of the dietitians provided nutrition care to COVID-19 patients, with 48.4% having access to personal protective equipment. Moreover, 17.0% of dietitians supported COVID-19 patients with enteral and parenteral nutrition. In addition, 45.0% of dietitians reported that managing COVID-19 was challenging given that it was a newly discovered condition.

## 1. Introduction

The unprecedented disturbance caused by COVID-19 has paralyzed the global and Arab health systems, putting immense pressure on the lives of healthcare workers [1]. Burnout has set in for healthcare professionals caring for COVID-19 patients as a result of their heavy workloads, the pandemic’s uncertainties, and the fear to carry the virus for their families and relatives [2]. The World Health Organization (WHO) estimates showed that 23–46% of healthcare workers reported anxiety symptoms during the COVID-19 pandemic, and 20–37% experienced depressive symptoms [3]. In addition, burnout among healthcare workers during the pandemic reached 41–52% in pooled estimates [3]. Early in the pandemic, 11% of reported cases of acute COVID-19 infection in the United States involved healthcare workers, and 8% of infected healthcare workers experienced a disease severe enough to necessitate hospitalization [4]. Besides, over 3600 healthcare workers died in the United States in the first year of the pandemic, according to a study from the Guardian Newspaper’s Lost on the Frontline Investigation [5]. Many healthcare professionals who were not directly providing care for COVID-19 patients faced being furloughed or having their hours shortened [6]. In May 2020, approximately 23% of non-hospital healthcare workers reported being unable to work at some point in the previous four weeks due to their employer closing or going out of business as a result of the pandemic, compared to 15% of hospital employees [6]. In the Arab world, one study aimed to investigate the COVID-19-related psychological impact on healthcare workers from 12 Arab countries and found that anxiety, depression, stress, and insomnia were identified in 48.9%, 50.6%, 41.4%, and 72.1% of respondents, respectively [7]. In addition, the prevalence of mental health problems was higher among healthcare workers who worked more than 44 h per week and those in contact with COVID-19 cases [7]. Besides, a systematic review showed that anxiety, depression, distress, stigmatization, risk of infection, and post-traumatic stress disorder (PTSD) were the most reported mental health issues among Arab health care workers [8]. On the other hand, the most reported physical health issues were physical abuse, exposure to the virus, exhaustion, burnout, underachievement, sleep deprivation, low life standard, and financial problems [8]. 

Dietitians, who serve as the foundation of any healthcare facility, have made significant contributions to the COVID-19 pandemic response [9]. Dietitians are specialists in determining the patient’s dietary needs while considering the patient’s age, gender, and any other underlying medical issues that may exist [10]. Nutrition is critical in assisting COVID-19 patients in fighting the infection and rapid recovery in the in-patient and out-patient settings [11]. COVID-19 patients could experience malnutrition and changes in eating habits and appetite, which would have a detrimental impact on their ability to recover and rehabilitate [11,12]. Dieticians’ management for obesity and other comorbidities also helps prevent severe COVID-19 complications, including acute respiratory distress syndrome (ARDS), intubation, severe pneumonia, and sepsis [13]. Thus, the role of dietitians in such nutrition-sensitive pandemic periods must not be underestimated. There are obvious differences in the roles and responsibilities of dietitians among countries. For example, most countries have nurses in intensive care units evaluate residual volume in the feeding tube after doctors administer enteral/parenteral nutrition. Different countries have different professionals who conduct screenings for malnutrition; in some, doctors do it, while in others, dietitians do. Fields of practice for dietitians in various countries are listed in Table 1. During the COVID-19 pandemic, dietitians have shouldered additional responsibilities that are deemed outside of their typical responsibilities, such as screening for COVID-19, and starting new duties in the Intensive Care Unit (ICU) [14]. According to a recent study [10], 40% of dieticians left their jobs due to the COVID-19 pandemic. Others claimed that trying to maintain a work-life balance while working in such a pandemic was the toughest for them [10]. Dietitians working in the ICU, in particular, had critical challenges to deal with [15]. Given the uncertain etiology of the coronavirus, the challenges may include establishing the optimum nutritional intervention to administer and managing a lack of nutrition support resources such as enteral feeds, syringes, and feeding tubes [15]. So far, studies investigating the impact of the COVID-19 pandemic on dietitians’ practices in their working facilities are scant worldwide. Thus, we launched this study in five Arab countries to assess the change in dietitians’ practices and duties in hospitals and clinics after the commencement of the COVID-19 pandemic with the associated challenges. 

## 2. Materials and Methods

### 2.1. Study Design and Data Collection

In respect to the study aims, a cross-sectional regional study was conducted over three months, from November 2020 to January 2021, in five Arab countries (Kuwait, Lebanon, Oman, Saudi Arabia, and Tunisia), when COVID-19 was an emergent pandemic worldwide. An online self-administered questionnaire was disseminated to be filled out by eligible dietitians. Participation calls were made via social media platforms and supported by the academic networks of the research team. The survey link was sent to dietitians through online applications or as email messages. To facilitate reaching dietitians, their contact information was obtained from ministries of health (MOH), non-governmental organizations (NGOs), and the alumni in each country. 

### 2.2. Dietitians’ Eligibility 

To be eligible for participation, dietitians should be working in hospitals or clinics in one of the mentioned Arab countries. Thus, an overall sample of 903 eligible dietitians completed the questionnaire, and their data were included in the analysis. 

### 2.3. Study Instruments

A questionnaire was developed by the study’s research team to collect relevant information from participants. The questionnaire was divided into multiple sections: the first section inquired about the demographic and professional characteristics of the dietitians (country of residence, age, gender, years of dietetic practice experience, highest education level, current employment status (full-time (≥40 h/week); part-time (<40 h/week)), and the practice facility (i.e., MOH public hospitals, university teaching hospitals, private hospitals, private clinics); (2) the second section investigated about the dietitians’ practices and duties in their facilities during COVID-19 and before the pandemic, this included: weekly workload compared to the pre-pandemic period (same, increased, decreased), caseload (number of patients with which the dietitian is concerned at one time) compared to the pre-pandemic period (same, increased, decreased), being given additional tasks that are deemed outside of the typical responsibilities with the type of the given tasks, change in the nutrition care provision mode (in-person or remote nutrition consultations), whether malnutrition assessment is routinely performed for patients in the hospital, providing nutrition care for patients in the ICU, and measuring the gastric residual volume (GRV) for tube-fed patients. The third section of the questionnaire gathered information about dietitians’ practices with regard to nutrition provision for COVID-19 patients. These practices included providing nutrition care (whether in-person or remotely) to patients who were confirmed or suspected to have COVID-19, having access to personal protective equipment (PPE), screening for malnutrition in COVID-19 patients with mentioning the tools used, the resources/guidelines used (and whether they are deemed adequate) when providing nutrition care to COVID-19 patients, methods used to calculate the energy requirements of COVID-19 patients, and other questions pertaining to details on the nutrition interventions and nutrition support (enteral nutrition (EN) and parenteral nutrition (PN)). Dietitians were also questioned about the challenges they frequently faced when providing nutrition care for COVID-19 patients in hospitals. 

### 2.4. Ethical Considerations 

The ethical committee at the University of Taibah, Saudi Arabia, had approved the current study protocol (reference: SREC/AMS 2020/65/CND). All dietitians provided written informed consent before filling out the questionnaire. Dietitians were welcome to contact the research team in each country with any questions about the study. There was no obligation to participate, and withdrawal was possible at all study stages. 

### 2.5. Data Analysis

All data were cleaned and exported to the Statistical Package of Social Sciences Software (SPSS) (Version 25.0. IBM Corp: Armonk, NY, USA) for analysis. The weighting of cases was performed to improve the representation of samples from each country. Frequencies (N) and percentages (%), were obtained for categorical variables, while means and standard deviations (SD) were used to summarize the findings of numerical variables. The chi-squared test (χ^2^) was used to determine the associations between study variables. In some circumstances, when one or more of the cell counts in a 2 × 2 table are less than 5, Fisher’s exact test was used instead. The McNemar’s test was also used to detect response differences between the two periods, before and during the COVID-19 pandemic. A *p*-value of 0.05 or lower was considered significant for all analytical tests. 

## 3. Results

### 3.1. Demographic and Profession-Related Characteristics of Dietitians Working in Hospitals and Clinics in Arab Countries

Table 2 shows the demographic and profession-related characteristics of dietitians working in hospitals and clinics in Arab countries. The practicing dietitians were recruited evenly from five Arab countries, as follows: Kuwait (20.0%), Lebanon (20.0%), Oman (20.0%), Saudi Arabia (20.0%), and Tunisia (20.0%). Almost half of the dietitians (48.8%) were in their 20s of age, whereas the half remaining (51.2%) were over 30 (the mean age ± SD of the overall sample was 31.8 ± 7.8 *years*). Female dietitians predominate in our sample (83.1%), with 16.9% being males. Nearly half (48.7%) of the dietitians had more than five years of experience in dietetic practice in hospitals and clinics. Around three quarters (71.0%) had a bachelor’s degree, 21.7% had a diploma, and 7.3% reported completing higher studies, holding master’s and doctorate degrees. Besides, 78.2% of the dietitians were committed to full-time work (≥40 h/week) in their facilities. The type of facilities they were working at were: Ministry of Health (MOH) public hospitals (60.4%), private hospitals (14.9%), university teaching hospitals (4.5%), military hospitals (2.5%), medical cities (1.5%), specialized hospitals (1.2%), national guard hospitals (0.3%). Others reported having their private clinics (8.8%) and working in dispensaries or polyclinics (5.9%). 

When stratified by their gender, we observed that the highest proportion of female dietitians was from Lebanon (23.8%), while male dietitians were mostly recruited from Saudi Arabia (39.4%), *p* < 0.001. Besides, female dietitians were significantly younger than their male counterparts (*p* = 0.02) and had fewer years of dietetic practice experience (*p* = 0.001). However, a significantly higher proportion of female dietitians (21.9%) had completed or pursued higher studies than males (20.5%), *p* = 0.01. Besides, more males than females were significantly committed to full-time work in their facilities (89.7% vs. 75.9%, *p* < 0.001). Although most dietitians were working in Ministry of Health (MOH) public hospitals, they were predominantly males (77.2% of male dietitians), *p* < 0.001.

### 3.2. Dietitians’ Practices and Duties in Hospitals and Clinics during the COVID-19 Pandemic in Arab Countries

Dietitians were asked to rate their workload (working hours per week) in their facilities in contrast to the pre-COVID period. Most dietitians (59.5%) reported that their workload did not change; however, 13.6% and 26.9% had their workload increased and decreased, respectively. The highest proportion of dietitians experiencing an increase in their workload was from Kuwait (35.3%), while 59.0% of dietitians working in Lebanon reported a workload decrease, *p* < 0.001. Besides, 19.8% and 24.2% of the overall dietitians stated that the caseload in their facilities had increased and decreased, respectively, during the pandemic. The caseload of dietitians had the highest increase among dietitians working in Saudi Arabia (44.1%), whereas it decreased significantly in Lebanon (48.5%) more than in other Arab countries, *p* < 0.001. In addition, a sizeable proportion of dietitians (18.7%) have been assigned additional jobs outside their institutions’ regular responsibilities. These were: screening for COVID-19 (5.5%), taking and recording patients’ temperatures (3.1%), covering out-patient clinics in addition to providing care to in-patients (3.6%), covering other wards outside of the usual routine (such as the ICU unit) (6.7%), inserting nasogastric feeding tubes (1.4%), performing food service tasks (i.e., tray line check) (8.4%), and other tasks (providing education sessions, preparing supplement formulas for patients) (2.2%). Dietitians working in Kuwait were assigned additional tasks in their facilities more than any other country (49.3%), *p* = 0.001. Moreover, in practice, after the onset of the COVID-19 pandemic, nearly half of the dietitians (46.9%) declared a change in the mode of nutrition care provided to patients, primarily those working in Saudi Arabia (76.3%). During the pandemic, the following modifications were made to nutrition care for patients: remote online nutrition consultation for all patients (20.0%), and remote online nutrition consultation only for suspected or confirmed COVID-19 patients (33.5%). Dietitians’ practices and duties in hospitals and clinics during the COVID-19 pandemic, overall and by country of residence were described in Table 3.

**Figure 1:**
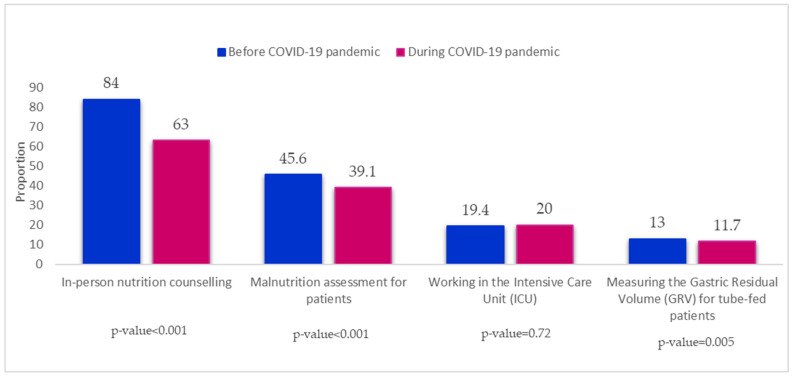
The alterations in dietitians’ practices in hospitals during the COVID-19 pandemic.

In Figure 1, further practice-related alterations are also depicted. The percentage of dietitians conducting in-person consultations with patients in hospitals or clinics has dropped when compared to the pre-pandemic period (pre-pandemic: 84.0% vs. post-pandemic: 63.0%, *p* = 0.001). Similar to this, a lower proportion of dietitians (39.0%) performed malnutrition assessment for patients during the pandemic as opposed to the pre-pandemic period (45.6%), *p* = 0.001. Additionally, fewer dietitians were used throughout the pandemic to assess the gastric residual volume (GRV) of patients receiving tube feedings (pre-pandemic: 13.0% vs. post-pandemic: 11.7%, *p* = 0.005). On the other hand, more dietitians provided nutrition care for critically ill patients in the ICU during the COVID-19 pandemic (20.0%) compared to the pre-pandemic period (19.4%), *p* = 0.72 (Figure 1).

### 3.3. Dietitians’ Practices in Providing Nutrition Care and Nutrition Support for COVID-19 Hospitalized Patients

Dietitians were asked about their nutrition care practices when dealing with COVID-19 patients. Among the 903 sampled dietitians, more than half (58.9%) reported providing nutrition care to patients with confirmed or suspected COVID-19. Of those, 34.0% provided in-person nutrition care to COVID-19 patients. Dietitians working in Oman (97.3%) and Saudi Arabia (78.5%) were more likely than those working in other Arab countries to report caring for COVID-19 patients (*p* = 0.001). Additionally, most dietitians working in Kuwait (64.2%) reported having direct contact with suspected and confirmed COVID-19 cases, *p* < 0.001. Nearly half (48.4%) of the dietitians claimed to have access to personal protective equipment (PPE) in their facilities, particularly in Kuwait (62.0%), *p* < 0.001. Overall, less than half the dietitians (38.3%) reported that they screened for suspected and confirmed cases of COVID-19 for malnutrition upon admission to the ICU, particularly in Oman (94.7% of the dietitians, *p* < 0.001). When asked about the tools used for malnutrition screening, the highest proportion of dietitians (30.2%) reported the use of the Malnutrition Universal Screening Tool (MUST), followed by the Nutrition Risk Screening 2002 (NRS 2002) (7.3%), the Mini Nutritional Assessment (MNA) (3.6%), and the Modified Nutrition Risk in Critically Ill (mNUTRIC) (2.7%) tools. American Society of Parenteral and Enteral Nutrition (ASPEN) (39.5%), World Health Organization (WHO) (39.2%), European Society of Clinical Nutrition and Metabolism (ESPEN) (37.1%), and country-specific Ministry of Health recommendations (32.0%) were rated as the most reliable resources when providing nutrition care to COVID-19 patients. These resources were used differently by dietitians in the Arab countries (*p*-values < 0.001).

Dietitians had also reported prescribing the following nutrition interventions for COVID-19 patients: oral nutrition prescription (16.7%), enteral and parenteral feedings (17.0%), nutrition supplement therapy (14.2%), nutrition education (12.7%), and nutrition counseling (10.1%). Furthermore, 17.0% of dietitians provided enteral or parenteral nutrition support for mechanically ventilated COVID-19 patients, particularly in Saudi Arabia (46.2% of dietitians), *p* = 0.01. As regards estimating the energy requirements for COVID-19 hospitalized patients, the participants indicated that the “Quick” method and the “Harris–Benedict equation” were the most used (10.7% and 6.2% of dietitians, respectively). Similarly, the energy requirements of COVID-19 patients were estimated differently in each Arab country (*p*-values < 0.001). Only 5.8% of the dietitians reported prescribing immune enhancer feeds or intravenous (IV) vitamins and trace elements for critically ill COVID-19 patients. Dietitians reported that inadequate energy, oral and protein intake were the most prevalent nutrition diagnosis among COVID-19 patients (reported by 14.0%, 13.3% and 9.9% of dietitians, respectively). Almost all the dietitians (96.0%) believed that their working facilities provide adequate resources for the nutritional management of COVID-19 patients, particularly in Oman and Tunisia (all dietitians were satisfied by the facilities’ resources, *p* < 0.001). Dietitians’ practices in providing nutrition care and nutrition support for COVID-19 hospitalized patients were described in Table 4.

### 3.4. Dietitians’ Experienced Challenges in Providing Nutrition Care for COVID-19 Patients in Arab Countries

**Figure 2:**
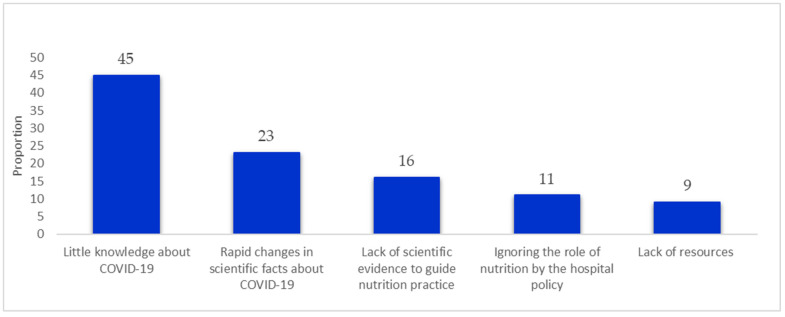
Dietitians’ experienced challenges in providing nutrition care for COVID-19 patients in Arab countries.

Nearly half the dietitians (45.0%) found it difficult to deal with COVID-19, given that it was an emerging disease with little knowledge about it. Other dietitians (23.0%) considered the rapid changes in the scientific facts about the disease as challenging because rapid changes in nutrition practice usually accompany this. Other reported challenges by dietitians included a lack of scientific evidence to guide nutrition practice (16.0%), ignoring the role of nutrition by the hospital policy (11.0%), and a lack of resources (9.0%) (Figure 2).

## 4. Discussion

This is one of the first cross-sectional observational studies to examine the immediate effects of the COVID-19 pandemic on the work of dietitians in hospitals and clinics, as well as the difficulties that have arisen as a result, in five Arab nations. Overall, nearly 40.0% of the dietitians experienced a change in their workload and caseload during the pandemic. Besides, 18.7% of the dietitians had been given additional tasks in their facilities, particularly food service tasks (8.4%). Moreover, 46.9% of the dietitians had started providing remote online nutrition consults, with 21.0% observing a drop in the percentage of dietitians conducting in-person consultations (*p* = 0.001). Additionally, during the pandemic, there was a decrease in the number of dietitians assessing malnutrition and taking GRV measurements for hospitalized patients (*p* = 0.001 and *p* = 0.005, respectively). Among all dietitians, 58.9% provided nutrition care to patients who confirmed or suspected to have COVID-19 (33.9% reported in-person care), with 48.4% having access to PPE in their facilities. The most used method for assessing malnutrition in COVID-19 patients was MUST. Additionally, 39.5% of the dietitians used ASPEN recommendations the most while providing nutrition care to COVID-19 patients. Besides, 17.0% reported supporting COVID-19 patients with enteral and parenteral nutrition. Among dietitians reporting to estimate the energy requirements of COVID-19 patients, the “Quick” method was the most used (10.7%). Just 5.8% reported prescribing immune enhancer feeds or intravenous vitamins and trace elements for critically ill COVID-19 patients. Almost all the dietitians (96.0%) believed that their working facilities provide adequate resources for the nutritional management of COVID-19 patients. On the other hand, nearly half the dietitians (45.0%) found it challenging to deal with COVID-19, given it was an emerging disease with little known about it.

In this study, a considerable proportion of dietitians experienced increased workloads and caseloads in their facilities; nonetheless, other dietitians had their duties decreased. According to the Academy of Nutrition and Dietetics [14], during the pandemic, many dietitians had been assigned additional responsibilities, including screening, treatment, and providing care for COVID-19 patients. This is also evident in the current study where we recorded that 18.7% of the dietitians had been given additional tasks in their facilities that are deemed outside of their typical responsibilities. On the other hand, a preliminary published study showed that many dietitians lost their jobs, had reduced working hours, experienced a change in the work environment, and shifted to remote and distanced consultations during the pandemic period [16]. This is also evidenced by a recent study by [10], who found that 40% of Emirati dietitians had quit their job since the start of the pandemic. In the current study, dietitians working in Lebanon reported having the highest decrease in their responsibilities and duties. It’s also important to note that Lebanon had one of the worst rates of unemployment in the region [17], which could have impacted the nutrition sector by keeping many dietitians out of work or working part-time. Lebanon’s unemployment rate increased from 11.4% in 2018–2019 to 29.6% in January 2022, revealing that almost one-third of the active labor force was unemployed in January 2022 [17]. 

The current study has also shown that 46.9% of dietitians have started giving remote nutrition consultations since the start of the COVID-19 pandemic. This confirms previous study findings showing that 72% of dietitians performed an alternative over-the- phone nutrition counseling during COVID-19, while 53.5% used an online platform [18]. It also comes in concordance with an Italian study showing that the proportion of registered dietitian nutritionists (RDNs) providing tele-nutrition had increased by 47% during the pandemic [19]. In this context, a recent systematic review showed that dietitians might improve nutrition outcomes for patients with or at risk of malnutrition [20]. Of relevance, previous studies have also observed significant malnutrition rates among COVID-19 patients [21,22]. Thus, remote nutrition consultation during pandemic periods may be the most effective means of ensuring the continuity of RDNs’ post-hospital nutritional therapy to reduce the nutritional effects of infection and promote at-home recovery. Remote communication made it possible to reach a greater segment of the population, particularly individuals who may have delayed visiting health services due to a fear of infection or a desire to reduce the burden on healthcare providers during the pandemic. As the COVID-19 pandemic decreases, these groups will require further help, as many may struggle to preserve their health and well-being [23]. However, some patients may benefit from virtual services, while others may not have a reliable phone or internet connection or the skills to use them [23]. Furthermore, people with deafness or hard of hearing may have trouble with virtual appointments, thus limiting their access to appropriate healthcare [23]. This increases health inequities for high-risk patients, such as older adults, low-income patients, and patients with disabilities [24,25,26]. Also, dietitians had limited-to-no ability to conduct a physical examination and relied on subjective and patient-reported measures (e.g., weight, appetite, or food intake) [23]. Different healthcare facilities, personnel, and specialties had poor communication and coordination [23]. Dietitians reported fewer referrals since the pandemic began in Canada, despite COVID-19’s nutritional hazards [23]. Preventing early intervention can induce malnutrition, weakness, and re-hospitalization [24,26]. As a result, it is recommended not to rely solely on remote consultations when providing individuals with health care but rather to utilize them as a supplement to in-person consultations and to follow up on patients’ cases when attendance stumbles.

In the current study, 58.9% of the dietitians reported providing nutrition care to confirmed or suspected COVID-19 patients, with 33.9% performing in-person care. Our findings corroborated data from the recently launched study in the UAE, where 65.8% of the dietitians stated counseling COVID-19 patients, and a third offered in-person consultations [10]. Nonetheless, 26.4% of Emirati dietitians reported not having access to PPE with half being worried about their safety and health [10]. This contradicts what we have observed, as 48.4% of the Arab dietitians currently assessed reported having access to PPE in their facilities. This was also manifested by the finding that almost all the dietitians (96.0%) were satisfied with the resources provided by their facilities for the nutritional management of COVID-19 patients. This is of particular relevance since a considerable proportion of the dietitians were providing in-person care to COVID-19 patients in the present study; the use of PPEs was fortunately prevalent among our sample. Evidence showed that properly using PPE can significantly lower the risk of infection associated with caring for COVID-19 patients [27]. Additionally, to reduce the risk of COVID-19 infection, a recent systematic review advocated the use of PPE by healthcare professionals, particularly N95 masks [28]. The availability of PPE, in addition to alternate remote consultations, enables continued nutrition therapy for COVID-19 patients while ensuring the dietitians’ safety. Ultimately, the role of nutrition in aiding COVID-19 patients to heal, recover faster, and avoid metabolic complications is indispensable and should be provided in safe and appropriate situations for all dietitians. Another finding of this study is that the most used method for assessing malnutrition in COVID-19 patients was MUST. Based on the observation of a cross-sectional study [29] demonstrating a possible use of MUST as a screening tool for malnutrition in COVID-19 patients, this finding is viewed as favorable. Moreover, a fortunate finding of this study is that 39.5% of the dietitians used ASPEN recommendations the most while providing nutrition care to COVID-19 patients. ASPEN had endorsed evidence-based recommendations concerning when to feed COVID-19 patients, the logistics of enteral feeding, meeting caloric and protein needs, selecting formulas and administering modes, monitoring tolerance to feeding, feeding while prone, and feeding during extracorporeal membrane oxygenation (ECMO) [30]. Also, in this study, enteral and parenteral nutrition was the most reported nutrition intervention to be prescribed for COVID-19 patients. This finding is warranted and justified. Due to the disturbance of the normal intestinal mucosa, patients with COVID-19 may have reduced nutrient absorption [30]. They frequently have considerable increases in their needs for protein and calories, as well as high levels of inflammation, stress, and catabolism [30]. To prevent malnutrition in these patients, immediate enteral nutrition support and the administration of adequate nutrients are crucial [31]. A recent systematic review and meta-analysis evidenced the effectiveness of enteral nutrition in alleviating the mortality risk among COVID-19 patients [31]. In the present study, the “Quick” method and the “Harris–Benedict equation” were the most used by dietitians to estimate the energy requirements of COVID-19 patients. Indirect calorimetry (IC) is the gold standard for determining energy expenditure in critically ill patients [32]. However, predictive equations can be utilized alternatively because IC is not always institutionally accessible [32]. Nevertheless, the risk of malnutrition, which is linked to poor clinical outcomes, is increased by the potential underestimation of energy requirements in severe COVID-19 patients [33]. 

The dietitians reported that inadequate energy, oral, and protein intake were prevalent among COVID-19 patients. Malnourished COVID-19 patients were observed previously to experience gastric intestinal distress, loss of smell, loss of taste, and shortness of breath [34]. Moreover, less than 39% of COVID-19 patients met the 1.2 g/kg/day ideal protein intake for recovery from illness [34]. Hence, our findings corroborated the published literature. Nearly half of the dietitians presently assessed found it challenging to deal with COVID-19, given that it was an emerging disease with little knowledge. Others were also challenged by the lack of scientific evidence to guide nutrition practice, ignoring the role of nutrition by the hospital policy, and the lack of resources. This comes hand in hand with a study showing that the uncertainty about the coronavirus impacted dieticians’ quality of life [16]. It is also consistent with the findings of a recent study among healthcare professionals who reported experiencing higher workloads, psychological distress, shortage of PPE, and stigmatization during the COVID-19 pandemic [35]. Furthermore, dietitians were particularly challenged during the COVID-19 pandemic with the necessity to prescribe nutrition supplements to COVID-19 patients because they were utilized over-the-counter, and manufacturers were marketing their products as COVID-19 preventatives or cures despite the lack of scientific proof for the effectiveness of many products [36]. Thus, they also were involved in educating the public about a plethora of nutrition-related misconceptions.

All in all, the results of our study showed that during the COVID-19 pandemic, dietitians in the Arab countries witnessed significant changes in their roles and responsibilities. These were manifested by changes in their workloads, caseloads, assigning new tasks in their facilities, and shifting to remote and distanced nutrition care provision. Besides that, they displayed crucial roles in the management of COVID-19 patients. However, their practices were somehow obstructed by many challenges. Dietitians are unsung medical heroes whose contributions shouldn’t be undervalued, particularly during nutritionally critical times like the COVID-19 pandemic. Almost all diseases, including the coronavirus, required “medical” and “nutrition” therapies. Hence, policymakers and decision-makers are encouraged to consider including dietitians in future training programs to help them cope resiliently with such pandemic periods with minimal withdrawals on their mental health and life quality.

### Study Limitations

The current study’s findings should be interpreted with caution due to some limitations. The study’s cross-sectional design allows for drawing associations between study variables with no causality. Furthermore, the online distribution of the questionnaire and the imposition of the restricted lockdown measures at the time of data collection made it impossible to have face-to-face communications with the study participants, raising the possibility of information bias in this study. 

## Figures and Tables

**Table 1:** Fields of practice for dietitians in various countries.

Country	Screening for Malnutrition	Assessing for Malnutrition	Prescribing of Nutritional Support	Formulation and Calculation Nutritional Support	Placing Feeding Tubes	Assessing of Residual Volume in Feeding Tube
Lebanon	Yes	Yes	No	Yes	No	Yes
Saudi Arabia	No	Yes	No	Yes	No	No
Kuwait	No	Yes	Yes	Yes	No	No
Oman	Yes	Yes	No	Yes	No	No
Tunisia	Yes	Yes	Yes	Yes	Yes	Yes

**Table 2:** Demographic and profession-related characteristics of dietitians working in hospitals and clinics in Arab countries.

	Overall (N = 903)		Females (n = 750)		Males (n = 153)		*p*-Value
	N	%	N	%	N	%	
Country of residence							<0.001 **
Kuwait	181	20.0	167	22.3	13	8.7	
Lebanon	180	20.0	178	23.8	2	1.2	
Oman	181	20.0	147	19.6	34	22.1	
Saudi Arabia	181	20.0	120	16.1	60	39.4	
Tunisia	180	20.0	136	18.2	44	28.6	
Age							0.02 *
20–30 years old	441	48.8	380	50.7	61	39.8	
>30 years old	462	51.2	370	49.3	92	60.2	
Years of dietetic practice experience							0.001 *
≤5 years	463	51.3	394	52.4	70	45.6	
More than 5 years	440	48.7	356	47.6	83	54.3	
Highest education level							0.01 *
Bachelor’s	641	71.0	521	69.5	119	77.9	
Diploma	196	21.7	64	8.5	2	1.6	
Master’s/Doctor of Philosophy (Ph.D.)	66	7.3	165	21.9	31	20.5	
Current employment status							<0.001 **
Full-time (≥40 h/week)	706	78.2	569	75.9	137	89.7	
Part-time (<40 h/week)	197	21.8	181	24.1	16	10.3	
Practice Facility							**<0.001**
Military hospitals	22	2.5	21	2.8	1	0.8	
Ministry of Health (MOH) public hospitals	545	60.4	427	57.0	118	77.2	
University teaching hospitals	41	4.5	39	5.2	2	1.1	
Specialized hospitals	11	1.2	7	0.9	4	2.3	
National guard hospitals	3	0.3	3	0.4	0	0.0	
Medical Cities	13	1.5	9	1.2	4	2.5	
Private hospitals	134	14.9	127	17.0	7	4.6	
Private clinics	80	8.8	76	10.1	4	2.3	
Others (dispensary, polyclinic)	53	5.9	40	5.3	14	8.9	

* Significant at *p*-value < 0.05 for χ^2^ test; ** Significant at *p*-value < 0.001 for χ^2^ test; Bold means significant for Fisher’s exact test.

**Table 3:** Dietitians’ practices and duties in hospitals and clinics during the COVID-19 pandemic, overall and by country of residence.

	Overall (N = 903)	Kuwait (n = 181)	Lebanon (n = 180)	Oman (n = 181)	Saudi Arabia (n = 181)	Tunisia (n = 180)	*p*-Value
	N (%)	n (%)	n (%)	n (%)	n (%)	n (%)	
Dietitians’ workload							<0.001 **
Similar to the pre-pandemic period	537 (59.5)	83 (46.0)	59 (32.5)	164 (90.7)	72 (39.8)	159 (88.3)	
Increased, compared to the pre-pandemic period	123 (13.6)	64 (35.3)	15 (8.5)	13 (7.3)	29 (16.1)	1 (0.6)	
Decreased, compared to the pre-pandemic period	243 (26.9)	34 (18.7)	106 (59.0)	4 (2.0)	80 (44.1)	20 (11.0)	
Dietitians’ case load							<0.001 **
Similar to the pre-pandemic period	403 (44.7)	41 (22.7)	41 (22.5)	155 (86.0)	41 (22.6)	125 (69.6)	
Increased, compared to the pre-pandemic period	179 (19.8)	70 (38.7)	12 (6.5)	16 (8.7)	80 (44.1)	2 (1.0)	
Decreased, compared to the pre-pandemic period	218 (24.2)	42 (23.3)	87 (48.5)	6 (3.3)	45 (24.7)	38 (21.0)	
The respondent is not aware	103 (11.4)	28 (15.3)	41 (22.5)	4 (2.0)1	16 (8.6)	5 (8.4)	
Being given additional tasks that are deemed outside of the typical responsibilities							<0.001 **
No	734 (81.3)	92 (50.7)	166 (92.0)	170 (94.0)	128 (71.0)	178 (98.7)	
Yes	169 (18.7)	89 (49.3)	14 (8.0)	11 (6.0)	52 (29.0)	2 (1.3)	
The type of the given task							
Screening for COVID-19	50 (5.5)	20 (11.3)	5 (3.0)	6 (3.3)	17 (9.7)	1 (0.3)	<0.001 **
Taking and recording patients’ temperatures	28 (3.1)	2 (1.3)	5 (3.0)	7 (4.0)	12 (6.5)	1 (0.6)	0.006 *
Started to cover out-patients’ clinics, in addition to providing care to in-patients	32 (3.6)	11 (6.0)	1 (0.5)	1 (0.7)	19 (10.8)	0 (0.0)	<0.001 **
Covering other wards outside of the usual routine	60 (6.7)	35 (19.3)	2 (1.0)	0 (0.0)	23 (12.9)	0 (0.0)	<0.001 **
Inserting nasogastric feeding tubes	13 (1.4)	6 (3.3)	1 (0.5)	0 (0.0)	6 (3.2)	0 (0.0)	<0.001 **
Performing food service tasks which not typically do (i.e., Tray line check)	76 (8.4)	58 (32.0)	4 (2.0)	1 (0.7)	14 (7.5)	0 (0.0)	<0.001 **
Other tasks (e.g., education sessions, preparing supplement formulas for patients)	20 (2.2)	5 (2.7)	3 (1.5)	0 (0.0)	12 (6.5)	1 (0.3)	<0.001 **
The mode of nutrition care provision to patients has changed at the facility							<0.001 **
No	480 (53.1)	57 (31.3)	75 (42.0)	152 (84.0)	43 (23.7)	153 (84.8)	
Yes	423 (46.9)	124 (68.7)	105 (58.0)	29 (16.0)	138 (76.3)	27 (15.2)	
The current mode of nutrition care provision for patients is (n = 423; among those who reported a change)							<0.001 **
Remote nutrition consults (for all patients)	120 (20.0)	44 (36.0)	35 (33.7)	0 (0.0)	39 (28.1)	1 (2.1)	
Remote nutrition consults (only for suspected or confirmed COVID-19 patients)	303 (33.5)	79 (64.0)	70 (66.3)	29 (100.0)	100 (71.9)	26 (97.9)	

* Significant at *p*-value < 0.05 for χ^2^ test; ** Significant at *p*-value < 0.001 for χ^2^ test.

**Table 4:** Dietitians’ practices in providing nutrition care and nutrition support for COVID-19 hospitalized patients.

	Overall (N = 903)	Kuwait (n = 181)	Lebanon (n = 180)	Oman (n = 181)	Saudi Arabia (n = 181)	Tunisia (n = 180)	*p*-Value
	N (%)	n (%)	n (%)	n (%)	n (%)	n (%)	
Providing nutrition care to patients who confirmed or suspected to have COVID-19							<0.001 **
No	371 (41.1)	53 (29.3)	120 (66.5)	5 (2.7)	39 (21.5)	155 (85.8)	
Yes	532 (58.9)	128 (70.7)	60 (33.5)	176 (97.3)	142 (78.5)	26 (14.2)	
Providing in-person nutrition care for COVID-19 patients (n = 532; among those reported providing care for COVID-19 patients)							
No	352 (66.1)	46 (35.8)	51 (81.4)	131 (75.3)	101 (71.2)	23 (90.9)	<0.001 **
Yes	180 (33.9)	82 (64.2)	12 (18.6)	43 (24.7)	41 (28.8)	2 (9.1)	
Having access to personal protective equipment (PPE) in your facility							<0.001 **
No	18 (2.0)	8 (4.7)	4 (2.0)	0 (0.0)	6 (3.2)	0 (0.0)	
Yes, most of the time	333 (36.9)	57 (31.3)	28 (15.5)	10 (5.5)	66 (36.6)	10 (5.5)	
Yes, occasionally	104 (11.5)	55 (30.7)	9 (5.0)	14 (7.8)	25 (14.0)	14 (7.8)	
Not applicable	448 (49.7)	60 (33.3)	140 (77.5)	156 (89.7)	83 (46.2)	156 (86.7)	
Malnutrition screening for suspected or confirmed COVID-19 patients							<0.001 **
No	557 (61.7)	116 (64.0)	155 (86.0)	10 (5.3)	99 (54.8)	178 (98.7)	
Yes	346 (38.3)	65 (36.0)	25 (14.0)	171 (94.7)	82 (45.2)	2 (1.3)	
Malnutrition screening tool							
MUST ^(a)^	272 (30.2)	53 (29.3)	8 (4.5)	171 (94.7)	39 (21.5)	1 (0.6)	<0.001 **
MNA ^(b)^	32 (3.6)	6 (3.3)	11 (6.0)	2 (1.3)	12 (6.5)	1 (0.6)	0.003 *
NRS2002 ^(c)^	66 (7.3)	4 (2.0)	9 (5.0)	24 (13.3)	29 (16.1)	0 (0.0)	<0.001 **
mNUTRIC ^(d)^	24 (2.7)	10 (5.3)	2 (1.0)	7 (4.0)	6 (3.2)	0 (0.0)	0.01 *
The resources/guidelines relied on when providing nutrition care to COVID-19 patients							
Country-specific Ministry of Health recommendations	289 (32.0)	71 (39.3)	39 (21.5)	51 (28.0)	107 (59.1)	22 (12.3)	<0.001 **
Country-specific Food and Drug Authority recommendations	67 (7.4)	8 (4.7)	15 (8.5)	4 (2.0)	33 (18.3)	7 (3.9)	<0.001 **
Country-specific Clinical Nutrition Society resources	38 (4.2)	5 (2.7)	4 (2.0)	14 (8.0)	16 (8.6)	0 (0.0)	<0.001 **
International Clinical Nutrition Society resources	105 (11.6)	13 (7.3)	9 (5.0)	65 (36.0)	17 (9.7)	0 (0.0)	<0.001 **
WHO recommendations	354 (39.2)	75 (41.3)	38 (21.0)	176 (97.3)	43 (23.7)	23 (12.9)	<0.001 **
ESPEN ^(e)^ guidelines	335 (37.1)	49 (27.3)	18 (10.0)	175 (96.7)	93 (51.6)	0 (0.0)	<0.001 **
ASPEN ^(f)^ guidelines	357 (39.5)	69 (38.0)	16 (9.0)	176 (97.3)	95 (52.7)	1 (0.3)	<0.001 **
Books	53 (5.9)	17 (9.3)	5 (3.0)	16 (8.7)	16 (8.6)	0 (0.0)	<0.001 **
Webinars	129 (14.2)	41 (22.7)	13 (7.0)	34 (18.7)	39 (21.5)	3 (1.6)	<0.001 **
Others	8 (8.8)	4 (2.0)	1 (0.5)	0 (0.0)	4 (2.2)	0 (0.0)	<0.001 **
The nutrition intervention provided for COVID-19 patients (n = 640)							
Oral nutrition prescription	151 (16.7)	49 (27.3)	14 (7.5)	8 (4.7)	80 (44.1)	0 (0.0)	<0.001 **
Nutrition support (EN or PN)	154 (17.0)	52 (28.7)	9 (5.0)	10 (5.3)	74 (40.9)	0 (0.0)	<0.001 **
Nutrition supplement therapy	129 (14.2)	47 (26.0)	6 (3.3)	1 (0.6)	66 (36.6)	9 (5.0)	<0.001 **
Nutrition education	115 (12.7)	47 (26.0)	10 (5.5)	10 (5.3)	45 (24.7)	1 (0.6)	<0.001 **
Nutrition counselling	91 (10.1)	27 (14.7)	12 (6.5)	11 (6.0)	41 (22.6)	1 (0.0)	<0.001 **
Most often provided nutrition support for mechanically ventilated COVID-19 patients							0.01 *
Generally, nutrition support is not provided	15 (1.6)	0 (0.0)	5 (3.0)	1 (0.7)	8 (4.3)	0 (0.0)	
Enteral nutrition	136 (15.0)	46 (25.3)	10 (5.5)	2 (1.3)	78 (43.0)	0 (0.0)	
Parenteral nutrition	18 (2.0)	7(4.0)	4 (2.0)	1 (0.7)	6 (3.2)	1 (0.3)	
Not sure	734 (81.3)	128 (70.7)	161 (89.5)	176 (97.3)	89 (49.5)	180 (99.7)	
Method used to estimate the energy requirements for COVID-19 patients (n = 272)							
Harris-Benedict equation	56 (6.2)	17 (9.3)	12 (6.5)	5 (2.7)	21 (11.8)	1 (0.6)	<0.001 **
Mifflin-St. Jeor equation	39 (4.3)	18 (10.0)	9 (5.0)	1 (0.7)	10 (5.4)	1 (0.3)	<0.001 **
Ireton-Jones equation	13 (1.4)	2 (1.3)	3 (1.5)	0 (0.0)	8 (4.3)	0 (0.0)	0.01 *
WHO/FAO/UNU	27 (2.9)	12 (6.7)	5 (3.0)	4 (2.0)	6 (3.2)	0 (0.0)	0.006 *
Schofield equation	9 (1.0)	2 (1.3)	3 (1.5)	2 (1.3)	2 (1.1)	0 (0.0)	0.610
TEE (total energy expenditure)	23 (2.5)	13 (7.3)	3 (1.5)	1 (0.7)	6 (3.2)	0 (0.0)	<0.001 **
The “Quick” method	97 (10.7)	22 (12.0)	7 (4.0)	2 (1.3)	64 (35.5)	1 (0.6)	<0.001 **
Others	14 (1.5)	2 (1.3)	4 (2.0)	0 (0.0)	8 (4.3)	1 (0.3)	
Prescribing immune enhancer feeds or intravenous vitamins and trace elements for critically ill COVID-19 patients							0.001 **
No	850 (94.2)	175 (96.7)	178 (99.0)	181 (100.0)	171 (94.6)	180 (99.7)	
Yes	53 (5.8)	6 (3.3)	2 (1.0)	0 (0.0)	10 (5.4)	1 (0.3)	
Most common nutrition diagnosis among COVID-19 patients							
Inadequate energy intake	127 (14.0)	53 (29.3)	9 (5.0)	2 (1.3)	82 (34.4)	0 (0.0)	<0.001 **
Inadequate oral intake	120 (13.3)	34 (18.7)	9 (5.0)	7 (4.0)	70 (38.7)	0 (0.0)	<0.001 **
Inadequate protein intake	89 (9.9)	47 (26.0)	6 (3.5)	0 (0.0)	10 (5.4)	1(0.3)	<0.001 **
Inadequate fluid intake	79 (8.7)	36 (20.0)	5 (2.5)	1 (0.7)	37 (20.4)	0 (0.0)	<0.001 **
Malnutrition (based on screening and assessment)	58 (6.5)	25 (14.0)	7 (4.0)	0 (0.0)	25 (14.0)	1 (0.3)	<0.001 **
Swallowing difficulties	54 (6.0)	18 (10.0)	6 (3.5)	10 (5.3)	19 (10.8)	1 (0.3)	<0.001 **
Altered gastro-intestinal function	52 (5.8)	16 (8.7)	4 (2.0)	0 (0.0)	33 (18.3)	0 (0.0)	<0.001 **
Unintended weight loss	51 (5.7)	27 (14.7)	7 (4.0)	0 (0.0)	17 (9.7)	0 (0.0)	<0.001 **
Overweight/obesity	44 (4.8)	14 (8.0)	1 (0.5)	1 (0.7)	27 (15.1)	0 (0.0)	<0.001 **
Feeding difficulties	43 (4.8)	16 (8.7)	1 (0.5)	1 (0.7)	23 (12.9)	0 (0.0)	<0.001 **
Inadequate enteral nutrition infusion	43 (4.7)	19 (10.7)	1 (0.5)	1 (0.7)	21 (11.8)	0 (0.0)	<0.001 **
Abnormal nutrition-related laboratory values	37 (4.1)	11 (6.0)	3 (1.5)	0 (0.0)	23 (12.9)	0 (0.0)	<0.001 **
Inadequate vitamin intake	37 (4.1)	22 (12.0)	3 (1.5)	0 (0.0)	12 (6.5)	1 (0.6)	<0.001 **
Inadequate mineral intake	32 (3.5)	20 (11.3)	1 (0.5)	0 (0.0)	10 (5.4)	1 (0.3)	<0.001 **
Excessive fluid intake	28 (3.1)	17 (9.3)	2 (1.0)	0 (0.0)	10 (5.4)	0 (0.0)	<0.001 **
Limited adherence to nutrition prescriptions	28 (3.1)	10 (5.3)	3 (1.5)	0 (0.0)	16 (8.6)	0 (0.0)	<0.001 **
Inadequate parenteral nutrition infusion	19 (2.1)	10 (5.3)	0 (0.0)	0 (0.0)	10 (5.4)	0 (0.0)	<0.001 **
Excessive fat intake	18 (2.0)	8 (4.7)	3 (1.5)	1 (0.7)	6 (3.2)	0 (0.0)	<0.001 **
Excessive carbohydrate intake	13 (1.4)	6 (3.3)	2 (1.0)	1 (0.7)	4 (2.2)	0 (0.0)	<0.001 **
The facility provides adequate resources for the nutritional management of COVID-19 patients							
No	36 (4.0)	4 (2.0)	2 (1.0)	0 (0.0)	31 (17.2)	0 (0.0)	<0.001 **
Yes	866 (96.0)	177 (98.0)	178 (99.0)	181 (100.0)	150 (82.8)	180 (100.0)	

^(a)^ MUST: Malnutrition Universal Screening Tool; ^(b)^ MNA: Mini Nutritional Assessment; ^(c)^ NRS 2002: Nutrition Risk Screening 2002; ^(d)^ mNUTRIC: Modified Nutrition Risk in Critically Ill; ^(e)^ ESPEN: European Society for Clinical Nutrition and Metabolism; ^(f)^ ASPEN: American Society for Parenteral and Enteral Nutrition; * Significant at *p*-value < 0.05 for χ^2^ test; ** Significant at *p*-value < 0.001 for χ^2^ test.

## Data Availability

Not applicable.
